# The Local Greying of Hair in Mice Treated with X Rays and Radiomimetic Drugs

**DOI:** 10.1038/bjc.1951.49

**Published:** 1951-12

**Authors:** E. Boyland, S. Sargent

## Abstract

**Images:**


### THE LOCAL GREYING OF HAIR IN MICE TREATED WITH X RAYS AND RADIOMIMETIC DRUGS

E. BOYLAND AND S. SARGENT.

*From the Chester Beatty Research Institute, Royal Cancer Hospital, London, S.W.3.*

Received for publication November 2, 1951.

THE exposure of living organisms to sublethal doses of radiation causes a number of changes, many of which can be also produced by radiomimetic drugs (Boyland, 1951). One of these is the local greying of the hair in mice described by Hance and Murphy (1926) and recently investigated by Chase (1949) following exposure to X rays. The same effect produced by implantation of plutonium has been described by Lisco, Finkel and Brues (1947). Chase (1949) considers that the measurement of the effect is suitable for the quantitative assay of biological effects of radiation. A very similar bleaching or greying of hair caused by injection of nitrogen mustard was described by Boyland, Clegg, Koller, Rhoden and Warwick (1948), and malignant tumours following nitrogen mustard treatment (in some cases in immediate contact with grey patches induced by the injection) have been described by Boyland and Horning (1949) and by Heston (1950).

The relationship between induction of grey hair and of cancer is investigated in the present paper. Localised greying of hair has now been induced in mice by a number of chemical agents which are known to be carcinogenic or mutagenic or both carcinogenic and mutagenic, or might be suspected of being so. The change is generally produced most readily by water-soluble substances but, with this limitation, the change might be used as a test indicating carcinogenicity. In this respect it is probably less specific, but similar to (1) the inhibition of tumour growth or body growth by carcinogenic compounds investigated by Haddow and his collaborators (Haddow, 1938), (2) the production of the specific chromosome damage described by Muller and Painter (1929) following X rays and by Darlington and Koller (1947) following mustard gas application, or (3) the increase in the mutation rate as described by Muller (1928) for X rays and by Auerbach, Robson and Carr (1947) for vesicants. The inhibition of growth and the induction of chromosome abnormalities in cells of small mammals can be observed readily with substances which are only very slightly soluble in water, but it is difficult to produce mutations in bacteria, *Neurospora* and *Drosophila* with substances which are not water-soluble.

Because the greying of hair described in the present paper is a discontinuous permanent change in a part of the soma, it had been suggested (Boyland, 1949) that it might be a somatic mutation and so analogous to the malignant change if the latter were a somatic mutation. The conception of somatic mutation in adult animals is, however, entirely hypothetical. If a mutation can only be proved by breeding and examination of the succeeding generations, then proof of the existence of mutations in somatic cells can never be established. The term "permanent modification" might be a more appropriate description. The local greying effect, however, is almost certainly due to selective destruction of dendritic cells producing pigment, and histological examination of affected hair follicles should give more information as to the nature of the change.

### EXPERIMENTAL.

Coloured mice (of either stock or pure lines C57 and C3H) were injected intradermally at one, two or four sites on the abdomen with aqueous solutions of the substances under investigation. In the tables, A refers to Agouti stock mice and B to black stock mice. The concentrations were such that the dose was contained in 0·05 ml. of solution. The mice were examined three times weekly for changes in the skin. If the substances were injected in aqueous solution they were only considered active if greying was seen in a higher proportion of mice than following injection of water. Other mice were exposed to a narrow beam of X rays for different times so that different doses of irradiation were given to limited areas. The X-ray tube was operated at 140 K.V.P., giving rays with a half value layer of 0·2 mm. copper and 140 r. per minute.

TABLE I.—*Greying of Hair in Mice Injected with Different Concentrations of Nitrogen Mustard and Related Carcinogenic Compounds.*

| Compound. | Concentration (mg./ml.). | Strain of mouse. | Number of mice. | Sites affected (%). | Mean latent period for appearance (days). |
|---|---|---|---|---|---|
| Methyl *bis* (β-chloroethyl)-amine (HN2) in water | 0·2 | CBA | 5 | 100 | 50 |
| | 0·2 | ABC | 24 | 100 | 60 |
| | 0·02 | Stock | 4 | 50 | 54 |
| | 0·002 | „ | 5 | 80 | 54 |
| Methyl *bis* (β-chloroethyl)-amine (HN2) in saline | 0·2 | B | 5 | 100 | 30 |
| | 0·1 | B | 5 | 100 | 25 |
| | 0·02 | B | 10 | 80 | 30 |
| | 0·002 | B | 4 | 50 | 47 |
| | 0·0002 | B | 10 | 10 | — |
| Methyl *bis* (β-chloroethyl)-amine and hyaluronidase in saline | 0·1 | B | 15 | 70 | 27 |
| β-Naphthyl *bis* (β chloroethyl)-amine (R 48) in arachis oil | 75·0 | C57 | 5 | 100 | 20 |
| *bis* (β-chloroethyl) sulphide (Mustard gas) in arachis oil | 0·4 | B | 20 | 60 | 56 |
| Mustard gas in propylene glycol | 0·4 | B | 10 | 90 | 24 |
| Mustard gas in water | 0·4 | B | 10 | 100 | 24 |
| 2:4:6-Tri (ethyleneimine) 1 : 3 : 5-triazine (R 246) in water | 0·0015 | CBA | 10 | 80 | 30 |
| 1:4-Dimethane sulphonoxy butane in arachis oil | 0·5 | Stock | 16 | 25 | 70 |
| 1:4-Dimethane sulphonoxy butane in saline | 2·0 | C57 | 5 | 5 | 40 |
| 1:6-Dimethane sulphonoxy hexane in arachis oil | 2·0 | Stock | 5 | 80 | 98 |
| *bis-p*-Toluene sulphonoxy-ethyl aniline in arachis oil | 20 | „ | 12 | 58 | 21 |
| 1:2:5:6-Diepoxyhexane in arachis oil | 5·0 | „ | 12 | 100 | 50 |
| 1:2:3:4:-Diepoxybutane in arachis oil | 2·5 | „ | 12 | 100 | 91 |
| 3:4-Benzpyrene in aqueous caffeine (2 per cent) | 0·05 | C57 | 10 | 0 | — |

Examples of greying produced by nitrogen and sulphur mustards are shown in Fig. 1 and 2. The data in Table I show that intradermal injection of a solution containing 0·002 mg. methyl *bis* ($\beta$-chloroethyl) amine hydrochloride (HN2) per ml. produced greying in half the treated animals, while injection of a more dilute solution produced the effect in only 10 per cent of animals. Thus the mean effective concentration is about 0·002 mg. per ml., equivalent to $10^{-5}$ M. For the purpose of rough quantitative comparison, the injected solution might be considered to be diluted twice in the skin so that the concentration in the tissue which evokes the effect is 0·001 mg. per ml. or $5 \times 10^{-6}$ M.

TABLE II.—*Greying of Hair of Stock Mice with Different Doses of X rays.*

| Dose (r). | Number of mice. | Mice affected. (%). | Latent period (days). | Mice affected on opposite side of body (%). | Latent period (days). |
|---|---|---|---|---|---|
| 50 | 2 | 0 | — | — | — |
| 100 | 2 | 0 | — | — | — |
| 200 | 2 | 0 | — | — | — |
| 250 | 4 | 0 | — | — | — |
| 400 | 2 | 0 | — | — | — |
| 500 | 6 | 33 | 60 | — | — |
| 800 | 6 | 50 | 188 | 50 | 213 |
| 1000 | 6 | 100 | 17 | 33 | 90 |
| 1500 | 2 | 100 | 40 | 100 | 120 |
| 1600 | 2 | 100 | 33 | 100 | 151 |
| 2000 | 1 | 100 | 40 | — | — |

The minimum dose of X rays which produced white hair in all cases (of Table II) was 1000 r (Fig. 3) and the median effective dose about 800 r. Thus irradiation with 800 r produces the same effect of depigmentation as $5 \times 10^{-6}$ M nitrogen mustard (HN2), so that in this reaction 100 r irradiation is equivalent to $0{\cdot}6 \times 10^{-6}$ M nitrogen mustard (HN2). In other radiomimetic effects, such as causing chromosome breaks and killing small animals, the equivalent doses have been considered to be $10^{-6}$ M nitrogen mustard and 100 r, which is of the same order as found with this new effect.

In the case of irradiated mice receiving more than 800 r greying was produced not only at the point of entry of the rays, but a secondary greying was induced at the point at which the rays left the body (Fig. 4). The absorption of radiation in the tissues would be such that the remote side of the body of the mouse would receive something over half of that given to the skin at the point of entry.

TABLE III.—*Greying of Hair Induced by Injection of Water into Mice.*

| Liquid injected. | Strain of mouse. | Number of mice. | Number of sites. | Sites affected (%). | Latent period (days). |
|---|---|---|---|---|---|
| Sterile distilled water | CBA | 12 | 48 | 8 | 40 |
| | FAKI | 6 | 24 | 33 | 30 |
| | C57 | 4 | 16 | 25 | 20 |
| | B | 67 | 228 | 24 | 20 |
| 0·9% sodium chloride | B | 40 | 160 | 0 | — |
| 0·9% sodium chloride acidified ph3 | Stock | 6 | 6 | 0 | — |
| 1*M* sodium chloride | ,, | 6 | 24 | 0 | — |
| 3 *M* sodium chloride | ,, | 10 | 10 | 40 | 50 |

Control experiments in the series with nitrogen mustard indicated that injection of water caused greying in a proportion of cases. The comparative effects of distilled water and saline are shown in Table III. Of the 46 mice injected intradermally with saline each at 4 sites, none showed greying, but of 70 mice

TABLE IV.—*Greying of Hair of Mice Injected with Substances which might Liberate Free Radicals and Related Compounds.*

Tetralin hydroperoxide, benzoyl peroxide and α-diphenyl-β-trinitro phenylhydrazyl were dissolved in arachis oil—other substances were in aqueous solution.

| Compound. | Molarity. | Strain of mouse. | Number of mice. | Number of sites. | Sites affected (%). | Latent period in days for appearance of 50% of maximum number of patches. |
|---|---|---|---|---|---|---|
| Hydrogen peroxide | 0·01 | B | 12 | 12 | 0 | — |
| | 0·1 | B and CBA | 34 | 58 | 12 | 54 |
| | 0·2 | B | 8 | 8 | 14 | 45 |
| | 0·5 | B | 2 | 8 | 37 | 19 |
| Hydrogen peroxide and ferrous sulphate | 0·01 | B | 5 | 5 | 20 | 50 |
| | 0·05 | A and CBA | 37 | 148 | 100 | 33 |
| | 0·1 | CBA | 25 | 38 | 32 | 40 |
| Ferrous sulphate | 0·05 | A and FAK | 10 | 25 | 88 | 30 |
| Ferric chloride | 0·01 | Stock | 10 | 40 | 60 | 27 |
| | 0·05 | ,, | 10 | 40 | 35 | 27 |
| Ascorbic acid and hydrogen peroxide | 0·01 | B | 8 | 8 | 12 | 44 |
| | 0·1 | C57 | 9 | 18 | 33 | 30 |
| Ascorbic acid | 0·01 | B | 8 | 8 | 12 | 40 |
| | 0·1 | C57 | 9 | 18 | 16 | 30 |
| Hydrogen peroxide and ultra-violet light | 0·5 | Stock | 12 | 48 | 25 | 23 |
| Ultra-violet light | — | ,, | 12 | 12 | 8 (slight) | 9 |
| Hydroxylamine and ferrous ammonium sulphate | 0·05 | ,, | 12 | 24 | 8 | 18 |
| Hydroxylamine | 0·05 | ,, | 12 | 12 | 8 | 32 |
| Ferrous ammonium sulphate | 0·05 | ,, | 12 | 12 | 41 | 20 |
| *tert.* Butyl hydroperoxide | 0·27 | ABC and stock | 27 | 108 | 51 | 25 |
| | 0·55 | Stock | 13 | 40 | 50 | 40 |
| Perbenzoic acid | 0·07 | C57 | 13 | 52 | 81 | 30 |
| Tetralin hydroperoxide | 0·036 | B | 7 | 7 | 0 | — |
| Benzoyl peroxide | 0·02 | B | 8 | 32 | 6 | 40 |
| | 0·04 | B | 6 | 6 | 1 | — |
| Diphenyl *bis* (diazonium hydroxide) | 0·01 | Stock | 8 | 32 | 32 | 26 |
| Benzene diazonium hydroxide | 0·02 | C57 | 15 | 60 | 81 | 60 |
| Sodium-*p*-benzene sulphonate diazonium hydroxide | 0·02 | Stock | 6 | 24 | 0 | — |
| α-Diphenyl-β-trinitro phenylhydrazyl | 0·01 | FAK IX | 5 | 20 | 0 | — |
| Azo-*bis*-isobutyronitrile in saline | 0·03 | B | 5 | 20 | 5 | 22 |

injected intradermally with distilled water, greying was produced at 19 per cent of the sites. The results suggest that the cellular change which causes the greying can result from exposure to hypotonic or hypertonic solutions.

Because the effects of radiation may be produced by liberation of free hydroxyl radicals within the cells, a number of reagents which might produce free radicals have been tested (Table IV). Free hydroxyl radicals can be produced by the interaction of hydrogen peroxide and reducing agents. The first agent of this type to be used was Fenton's reagent, made by mixing hydrogen peroxide and ferrous sulphate, which react together very rapidly. Because of the rapidity of reaction the solutions were injected separately. The solution of ferrous sulphate (0·05 ml.) was injected and then an equal volume of hydrogen peroxide injected at the same site. The injection of hydrogen peroxide alone induced about the same degree of greying as injection of water, but the injection of ferrous sulphate either with or without hydrogen peroxide caused greying in most cases. The induction of the effect with ferrous sulphate and hydrogen peroxide suggested

TABLE V.—*Greying of Hair of Mice Injected with Various Compounds, including Carcinogens (C) and Substances which produce Mutations (M), Chromosome Damage (D) or Blisters (B).*

| Compound. | Solvent. | Concentration (mg./ml.). | Strain of mouse. | Number of mice. | Number of sites. | Sites affected. (%). | Latent period (days). |
|---|---|---|---|---|---|---|---|
| Benzidine (C) | N/40 HCl | 1·8 | Stock | 6 | 24 | 20 | 25 |
| 3:3'-Dihydroxybenzidine (C) | Arachis oil | 10 | ,, | 35 | 140 | 6 | 78 |
| | Water | 35 | B | 10 | 40 | 12·5 | 26 |
| 2'-Chloro-4-dimethylaminostilbene (C) | Arachis oil | 12·5 | CBA | 10 | 40 | 0 | — |
| 2'-Fluoro-4-dimethylaminostilbene (C) | Ditto | 50 | CBA | 9 | 36 | 0 | — |
| 4'-Fluoro-4-dimethylaminostilbene (C) | ,, | 25 | B | 11 | 44 | 2 | 113 |
| 2-Amino-1-naphthol (C) | ,, | 10 | B | 5 | 20 | 10 | 120 |
| Benzoquinone (C) | Water | 0·8 | ABC | 8 | 8 | 62 | 69 |
| Beryllium chloride (C) | ,, | 50 | C57 | 7 | 28 | 0 | — |
| | | 10 | C57 | 8 | 32 | 0 | — |
| Sodium arsenite (C) | ,, | 1·0 | C57 | 8 | 32 | 0 | — |
| Urethane and ultra-violet light (C) | ,, | 100 | Stock | 11 | 48 | 12 | 30 |
| | ,, | 50 | ,, | 4 | 16 | 8 | 28 |
| Urethane (C) | ,, | 100 | Mixed | 5 | 20 | 5 | 52 |
| | ,, | 50 | ,, | 15 | 60 | 3 | 52 |
| Formaldehyde (M) | ,, | 2·0 | C57 | 19 | 76 | 52 | 63 |
| Pyridine-3-diethylcarbonamide (B) | ,, | 5·0 | B | 9 | 36 | 19 | 13 |
| Aminopterin (D) | ,, | 0·5 | B | 8 | 8 | 0 | — |
| Streptomycin (M ?) | ,, | 50 | B | 5 | 5 | 0 | (Kept |
| | ,, | 25 | B | 5 | 5 | 0 | 48 days) |
| Cortisone | Saline | 10 | B | 5 | 20 | 0 | — |
| β-Propiolactone (B) | ,, | 0·75 | C57 | 10 | 40 | 0 | — |
| Lanthanum acetate | Water | 20 | FAK IX | 4 | 16 | 87 | 40 |

that free hydroxyl radicals might be the cause of the change, but the action might be due to the iron salt alone acting as catalysis of oxidation processes possibly involving free radicals, or to direct chemical combination with some cell constituent such as nucleic acid.

Exposure of mice injected with hydrogen peroxide to ultra-violet light (5 minutes' exposure at 3 cm. distance from an Osira lamp) did not cause a significant increase in the incidence of greying. The penetration of the ultra-violet light was probably insufficient to produce free hydroxyl radicals from the injected peroxide. The injection of urethane with or without exposure to ultra-violet light also had no significant greying effect (Table V).

Combined treatment with hydroxylamine and ferrous ammonium sulphate was tested because the reagents might react to produce free amine ($NH_2$) radicals. The hydroxylamine appeared to reduce the effect of the iron salt.

Of the organic peroxides tested *tert.* butyl hydroperoxide and perbenzoic acid were effective. Of these *tert.* butyl hydroperoxide is known to produce free radicals and has produced mutations in *Neurospora* (Dickey, Cleland and Lotz, 1949).

Of other substances which might yield free radicals listed in Table IV, benzene diazonium hydroxide caused considerable greying. On the other hand, sodium diphenyl *bis* diazonium hydroxide (diazotised benzidine) and sodium *p* benzene sulphonate diazonium hydroxide (diazotised sulphanilic acid) were inactive. The inactivity of these two diazo compounds may be due to inability to penetrate to the cell nucleus before they have reacted or decomposed.

Of the substances in Table V which were inactive, some have been described as being carcinogenic, including the dimethyl-aminostilbene derivatives, which are almost insoluble in water (Haddow, Harris, Kon and Roe, 1948) and 3:3′-dihydroxy-benzidine (Baker, 1950), while others which are mitotic poisons include aminopterin (Dustin, 1950) and β-propiolactone. Other inactive substances were pyridine-3-diethylcarbonamide which has been described as vesicant (Carlsson and Serin, 1949), cortisone which like aminopterin has a leucopenic action, and streptomycin which may increase the incidence of mutations in bacteria.

The activity of formaldehyde is of interest in view of the reported mutagenic action of this substance (Auerbach, 1950; Rapaport, 1946). Benzoquinone, which caused greying, has been described as carcinogenic (Takizawa, 1940). The benzyl ether of hydroquinone which causes leucoderma in negroes (Schwartz, Oliver and Warren, 1940) and 3:4-dimethylphenylthiourea which inhibits phenoloxidases (Jaques, 1950) did not cause greying. The 2-amino-1-naphthol, a tumour metabolite of β-naphthylamine described as carcinogenic by Hueper (1938), produced slight greying when injected in oil.

The greying was also induced by application of cold in the form of carbon dioxide snow. When small pieces of carbon dioxide were held on the fur of mice no effect was produced. The application of the carbon dioxide snow to the skin of plucked mice for two minutes produced white patches in each of five mice, within 19 days of the application. Taylor (1949) had observed a similar effect on melanophores of rats, and Berenblum (1930) induced tumours in mice by this treatment.

The loss of colour of the hair might be due to deficiency of one or more enzymes necessary for melanin formation in the skin. For this reason dihydroxyphenylalanine (" dopa "), an intermediate in the process, was injected intradermally into white patches induced by nitrogen mustard to see whether it could reverse the process. Five black mice bearing clear white patches were used. The sizes

---

EXPLANATION OF PLATES.

FIG. 1.—Black Stock mouse, 4 months after a single intradermal injection of HN2 (0·1 mg./kg.). The white hairs appeared 3 weeks after injection.

FIG. 2.—Black Stock mouse, 10 months after a single intradermal injection of β:β′-dichlorodiethyl sulphide (4 mg./kg. mustard gas). In this mouse also the white hairs appeared three weeks after injection.

FIG. 3.—Black Stock mouse, 2 months after local irradiation with 1000 r.

FIG. 4.—Black Stock mouse (photographed on mirror so that underside can be seen) 10 months after irradiation with 1600 r, showing white patches at points of entry and exit of X rays. The primary patch on the back appeared after 2 months.

1

2

3

4

and shapes of the patches were noted, and then all the white hair and some of the surrounding black hair was plucked out. The mice were injected with 2 per cent " dopa " solution the following day when the hair should be in an actively growing phase. After 10 days the white hair could be seen growing again, and in each case it eventually showed the same distribution as before.

The injection of nitrogen mustard into the skin of coloured guinea-pigs produced scars, the surrounding edge of which had white hairs. This is comparable with the scar production seen after irradiation of guinea-pigs by Frederic (1949) but since he used white animals, there is no record of colour change. A black rabbit was also injected with nitrogen mustard, but no greying was seen in this animal, although it showed scarring similar to that in the guinea-pigs.

### DISCUSSION.

The greying of hair in mice is a radiomimetic effect, the significance of which is difficult to assess. The production of the change by injection of distilled water suggests that the effect of hypotonic solutions on induction of chromosome abnormalities, mutations and other changes should be examined. Although the change is produced by water-soluble carcinogenic agents, there are some substances which have produced greying which have not as yet been shown to be carcinogenic. But as it is hoped that the effect might be used to reveal hitherto unsuspected carcinogenic stimuli, some of these agents are being tested for carcinogenic action. The process of carcinogenesis is obviously complex, and some substances may only have the initiating action and none of the developing or co-carcinogenic action.

Some chemically reactive substances, such as the mineral acids and trichloracetic acid kill cells so that specific biological changes are not produced by such agents, but only by substances with limited toxic action. Although some vesicants are mutagenic and carcinogenic, lewisite, which is a powerful vesicant, does not appear to be mutagenic, and many carcinogens are not vesicant, so that the effects are not always associated. The association between induction of mutations and induction of cancer is much closer.

The effect of local greying resembles the induction of specific chromosome abnormalities, of mutations of cancer and of blisters on human skin in that it is produced by agents with limited toxic action. In the case of the agents producing chromosome changes, mutations or cancer the effect is limited to specific parts of cells. In the local greying of hair the effect is restricted to certain specific cells of the tissue.

### SUMMARY.

(1) The intradermal injection of nitrogen mustards or sulphur mustard into coloured mice causes a permanent greying or bleaching of the hair similar to that occurring after exposure to ionising radiations. This greying appears to be a radiomimetic effect.

(2) Similar greying of hair occurs in a proportion of cases when water is injected but never following the injection of saline.

(3) Greying of hair was produced by injection of (*a*) known carcinogens, such as $\beta$-naphthyl-*bis* ($\beta$ chloroethyl) amine and butadiene diepoxide, the mustards, 2:4:6-tri(ethyleneimino) 1:3:5-triazine and benzoquinone, (*b*) known cytotoxic agents and mutagens which are not yet known to be carcinogenic, such as *tert.* butyl-hydroperoxide and formaldehyde, (*c*) substances which are not known to be carcinogenic, cytotoxic or mutagenic, including salts of iron and lanthanum and substances which might give free radicals, such as benzene diazonium hydroxide and perbenzoic acid.

(4) Greying was not produced by injection of some carcinogens, including urethane and dimethylaminostilbene derivatives.

(5) This effect, which may be an induced permanent variation due to preferential lethal action on pigment-producing dendritic cells, is an example of limited toxic action, and so allied to vesication and induction of mutations and cancer.

We should like to thank Miss Harriss and Dr. Lamerton for help in the irradiation experiments, Mr. F. Speed for the photographs and Mr. J. A. Marsh for his assistance.

This investigation has been supported by grants to the Royal Cancer Hospital and the Chester Beatty Research Institute from the British Empire Cancer Campaign, the Anna Fuller Fund, the Jane Coffin Childs Memorial Fund, and the National Cancer Institute of the National Institutes of Health, U.S. Public Health Service.

### REFERENCES.

AUERBACH, C.—(1950) Paper at Symp. on Biochem. Genetics, London.
*Idem*, ROBSON, J. M., AND CARR, J. G.—(1947) *Science*, **105**, 243.
BAKER, K.—(1950) *Acta unio int. contra cancrum*, **7**, 46.
BERENBLUM, I.—(1930) *Brit. J. exp. Path.*, **11**, 208.
BOYLAND, E.—(1949) *Pontif. Acad. Sci. scripta varia*, **7**, 79.—(1952) *Endeavour*, **11**, 70.
*Idem*, CLEGG, J. W., KOLLER, P. C., RHODEN, E., AND WARWICK, O. H.—(1948) *Brit. J. Cancer*, **2**, 17.
*Idem* AND HORNING, E. S.—(1949) *ibid.*, **3**, 118.
CARLSSON, A., AND SERIN, F.—(1949) *K. fysiogr. Sällsk. Lund. Förh.*, **19**, 233.
CHASE, H. B.—(1949) *J. Morph.*, **84**, 57.
DARLINGTON, C. D., AND KOLLER, P. C.—(1947) *Heredity*, **1**, 187.
DICKEY, F. H., CLELAND, G. H., AND LOTZ, C.—(1949) *Proc. nat. Acad. Sci., Wash.*, **35**, 581.
DUSTIN, P.—(1950) *Revue d'Hematologie*, **5**, 603.
FREDERIC, J.—(1949) *Arch. Biol., Paris*, **60**, 79.
HADDOW, A.—(1938) *J. Path. Bact.*, **47**, 372.
*Idem*, HARRIS, R. J. C., KON, G. A. R., AND ROE, E. M. F.—(1948) *Trans. Roy. Soc.*, A, **241**, 147.
*Idem* AND TIMMIS, G.—(1950) *Int. Congrès Cancer*, **5**, 90.
HANCE, R. T., AND MURPHY, J. B.—(1926) *J. exp. Med.*, **44**, 339.
HESTON, W. E.—(1950) *J. nat. Cancer Inst.*, **11**, 415.
HUEPER, W. C.—(1938) *Arch. Path.*, **25**, 856.
JAQUES, R.—(1950) *Helv. Chim. Acta*, **33**, 650.
LISCO, H., FINKEL, M. F., AND BRUES, A. M.—(1947) *Radiology*, **49**, 301.
MULLER, H. J.—(1928) *Proc. nat. Acad. Sci., Wash.*, **14**, 714.
*Idem* AND PAINTER, E. E.—(1929) *J. Heredity*, **20**, 287.
RAPAPORT, I. A.—(1946) *C. R. Acad. Sci. U.R.S.S.*, **54**, 65.
SCHWARTZ, L., OLIVER, E. A., AND WARREN, L. H.—(1940) *Publ. Hlth. Rep.*, **55**, 1111.
TAKIZAWA, N.—(1940) *Proc. imp. Acad. Japan*, **16**, 309.
TAYLOR, A. C. J.—(1949) *J. exp. Zool.*, **110**, 77.


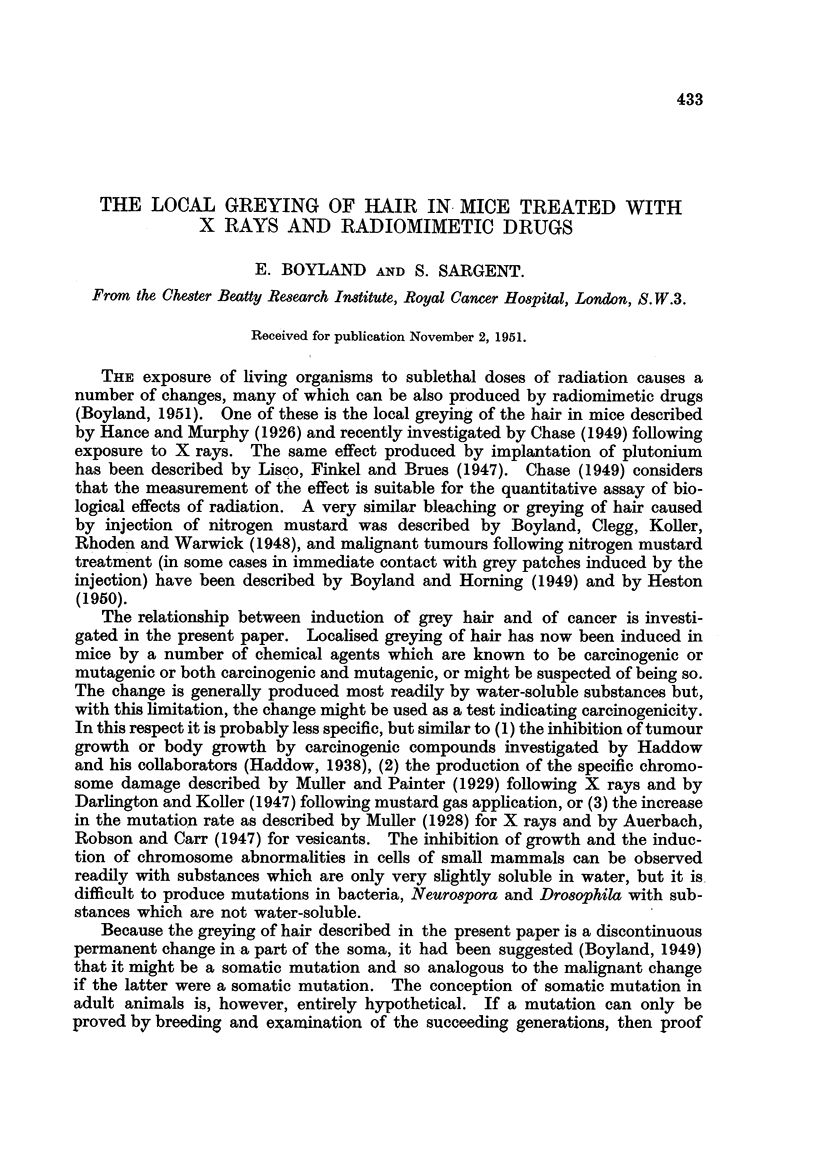

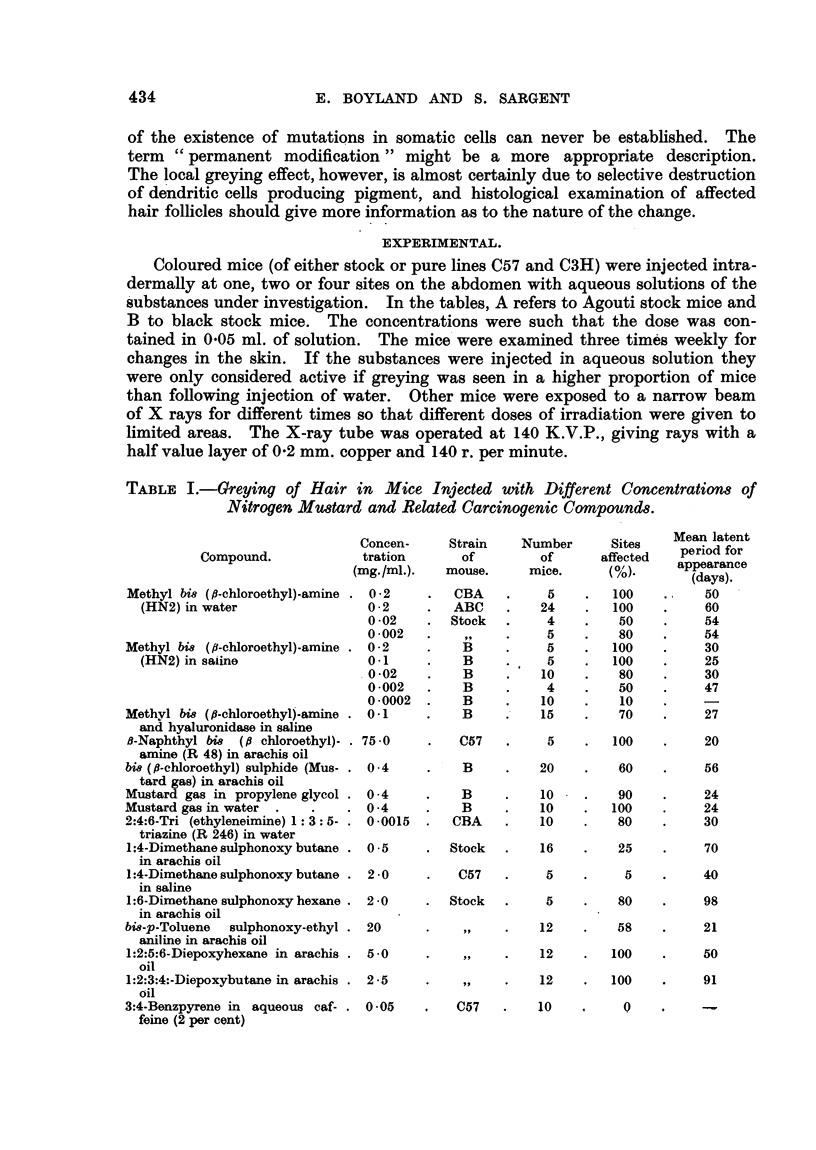

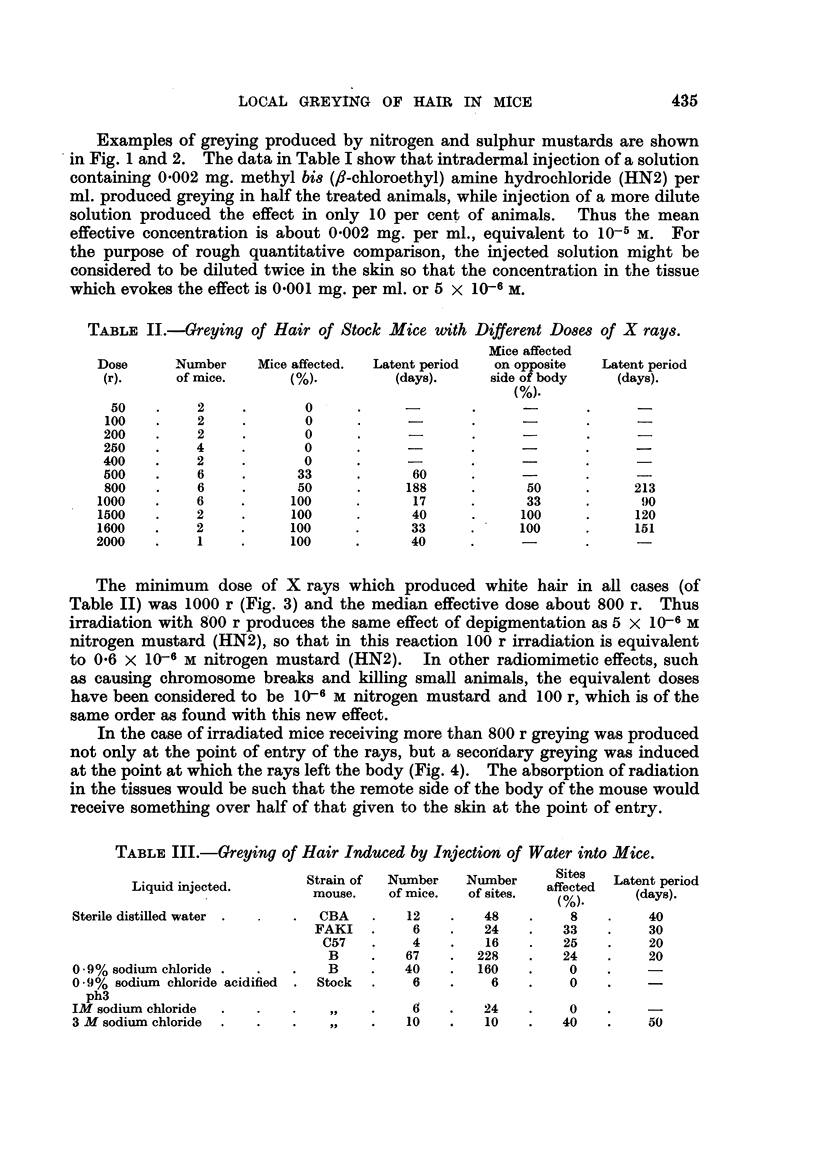

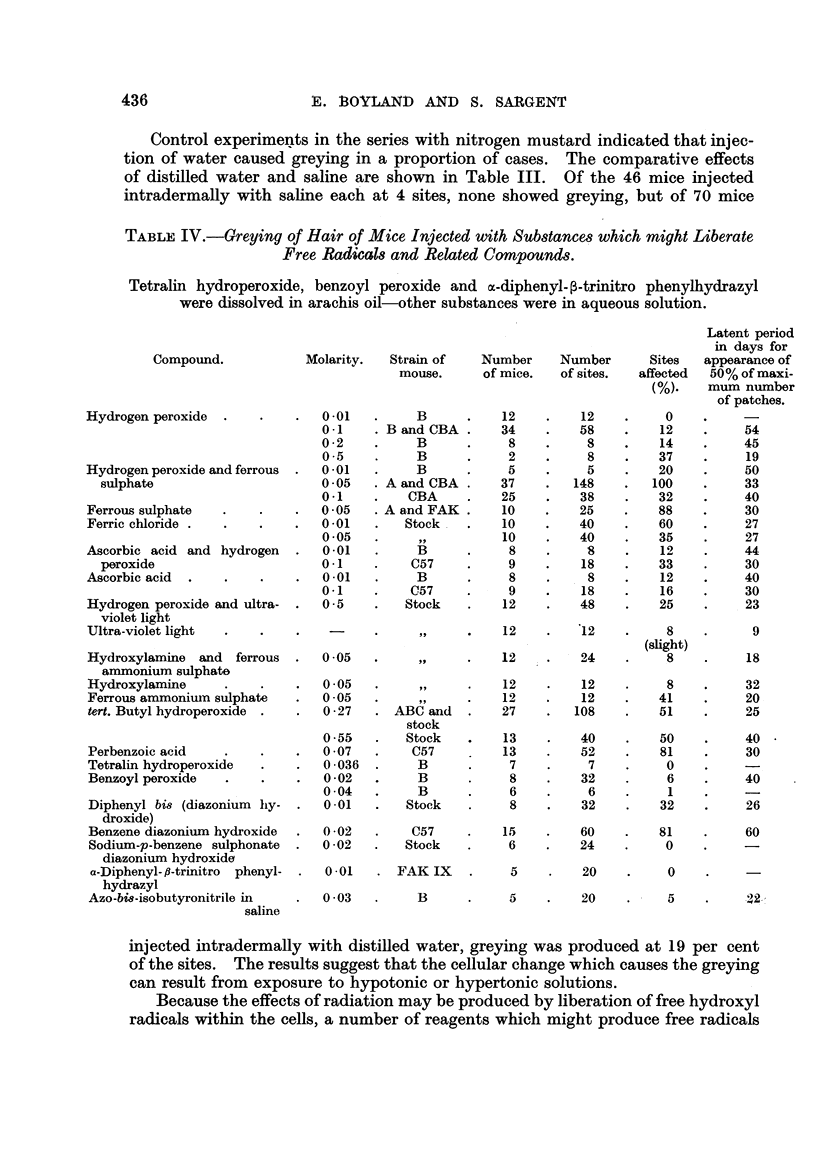

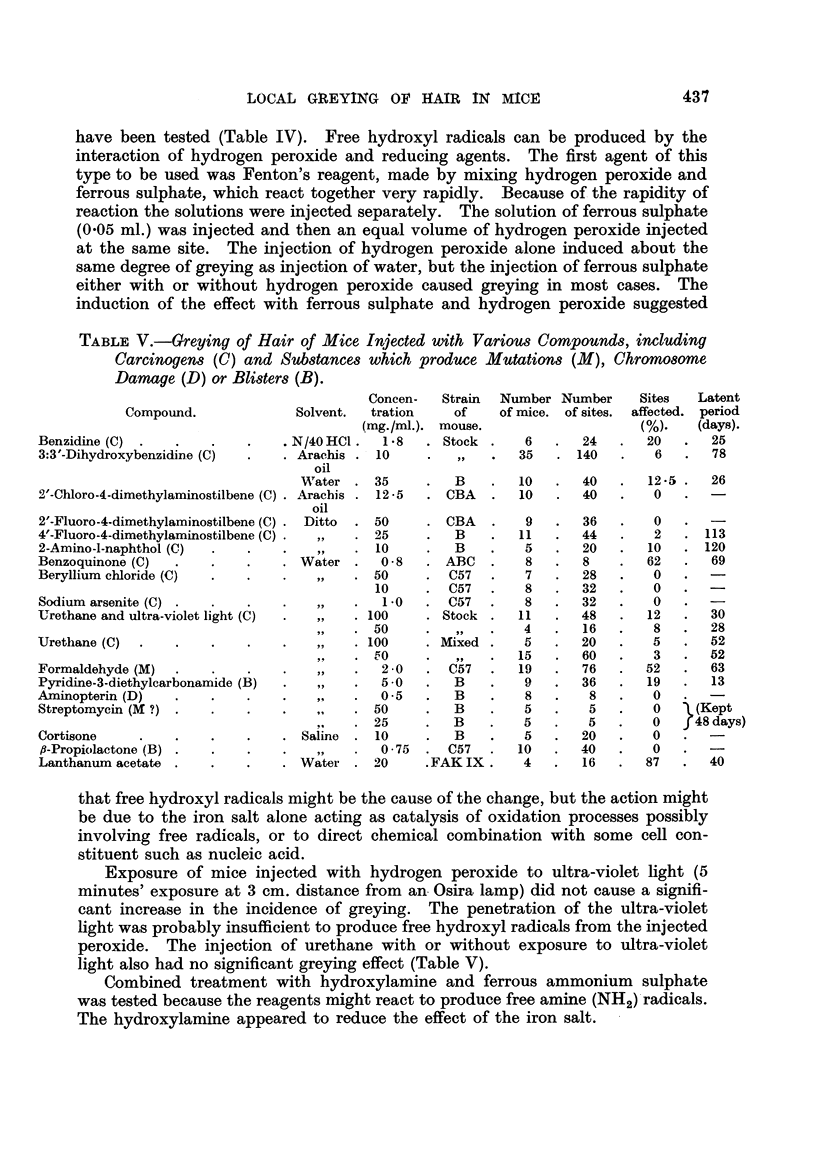

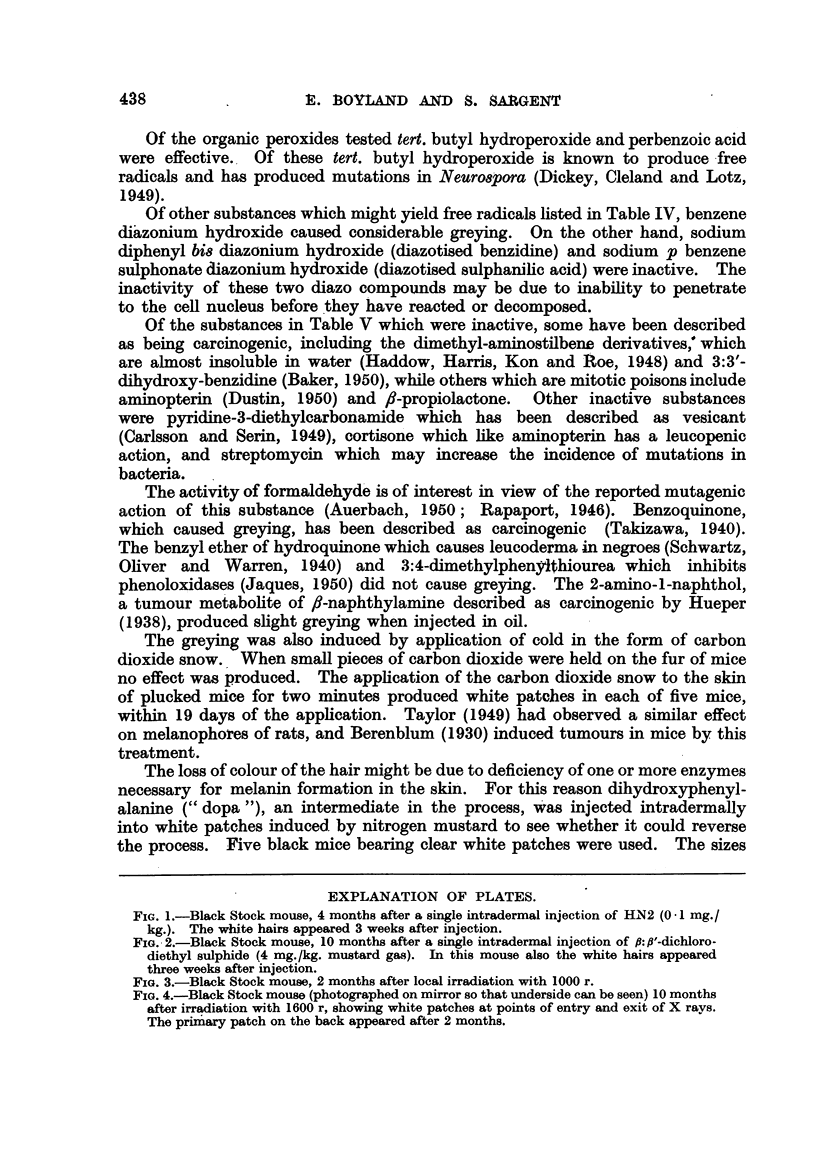

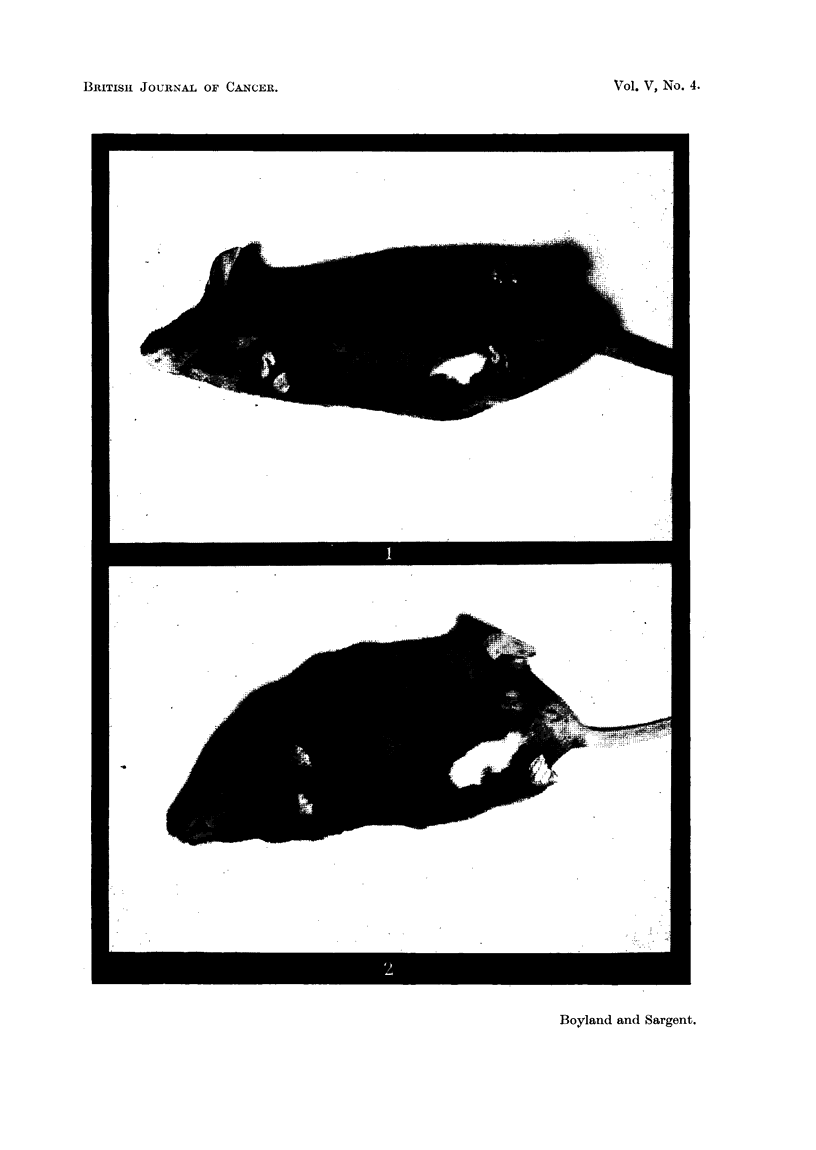

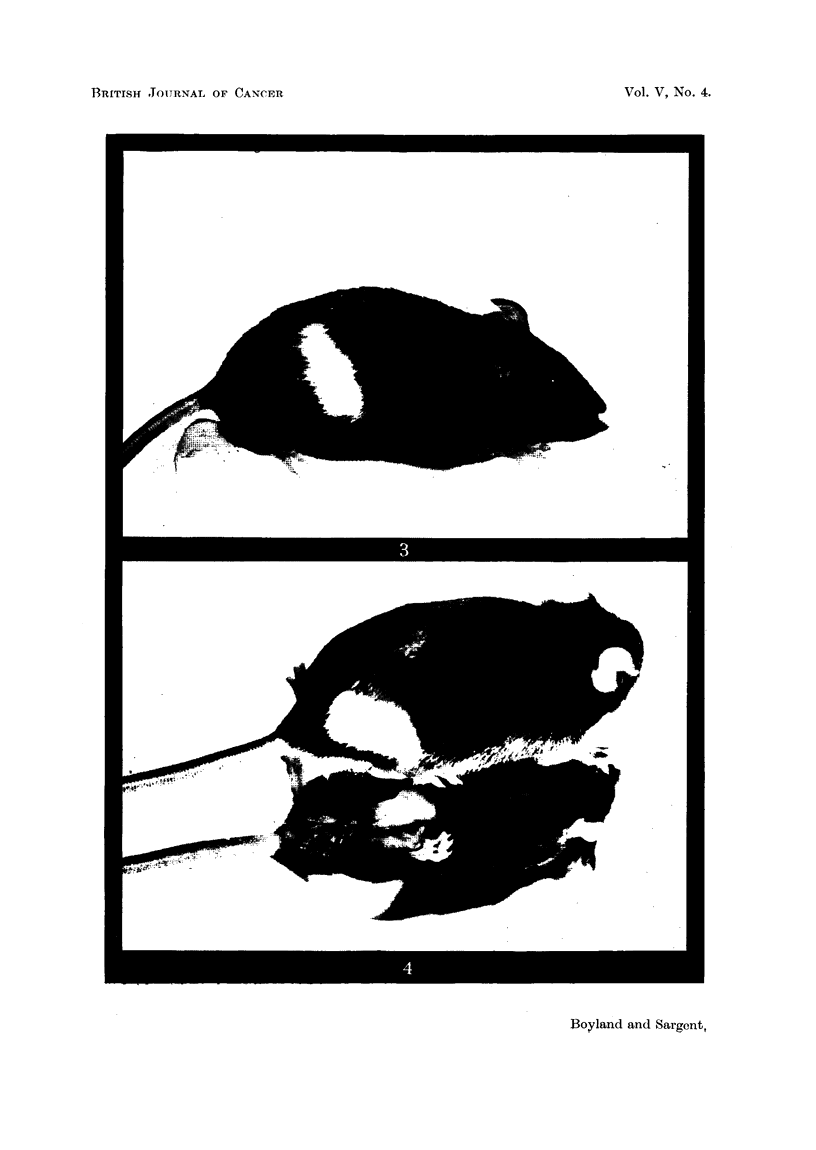

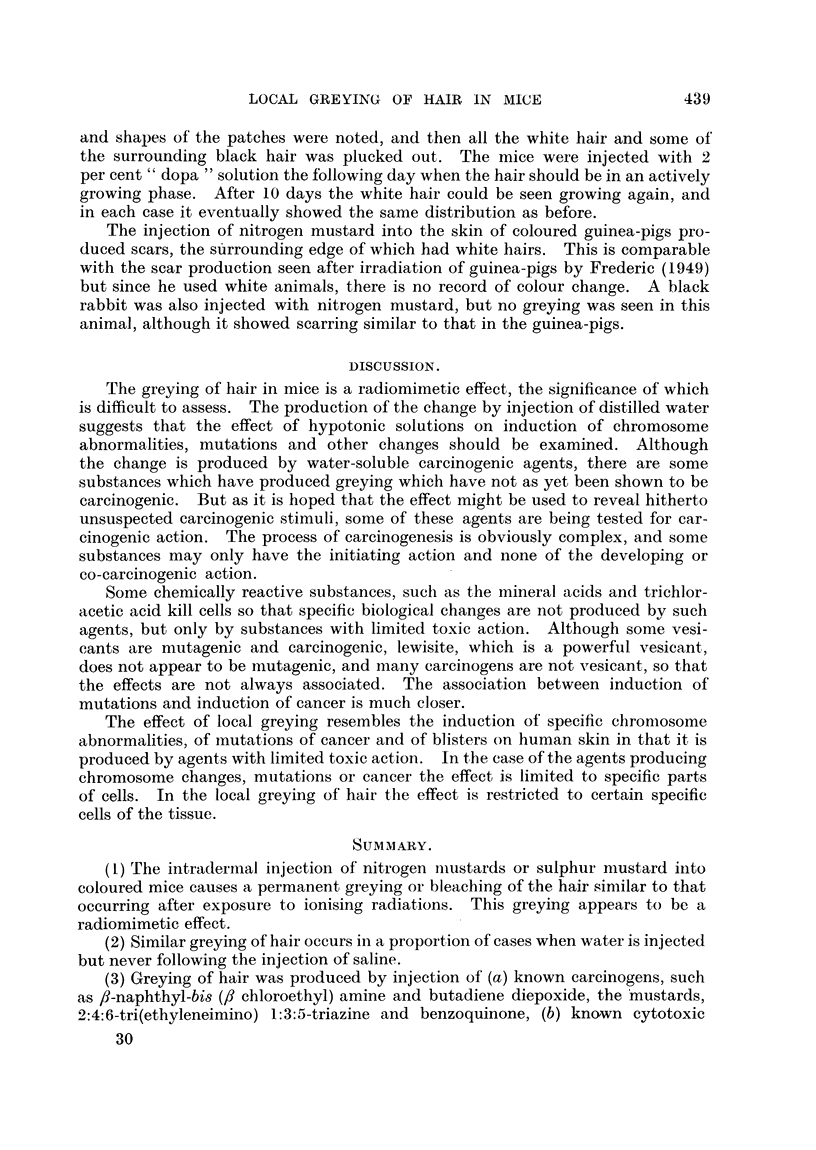

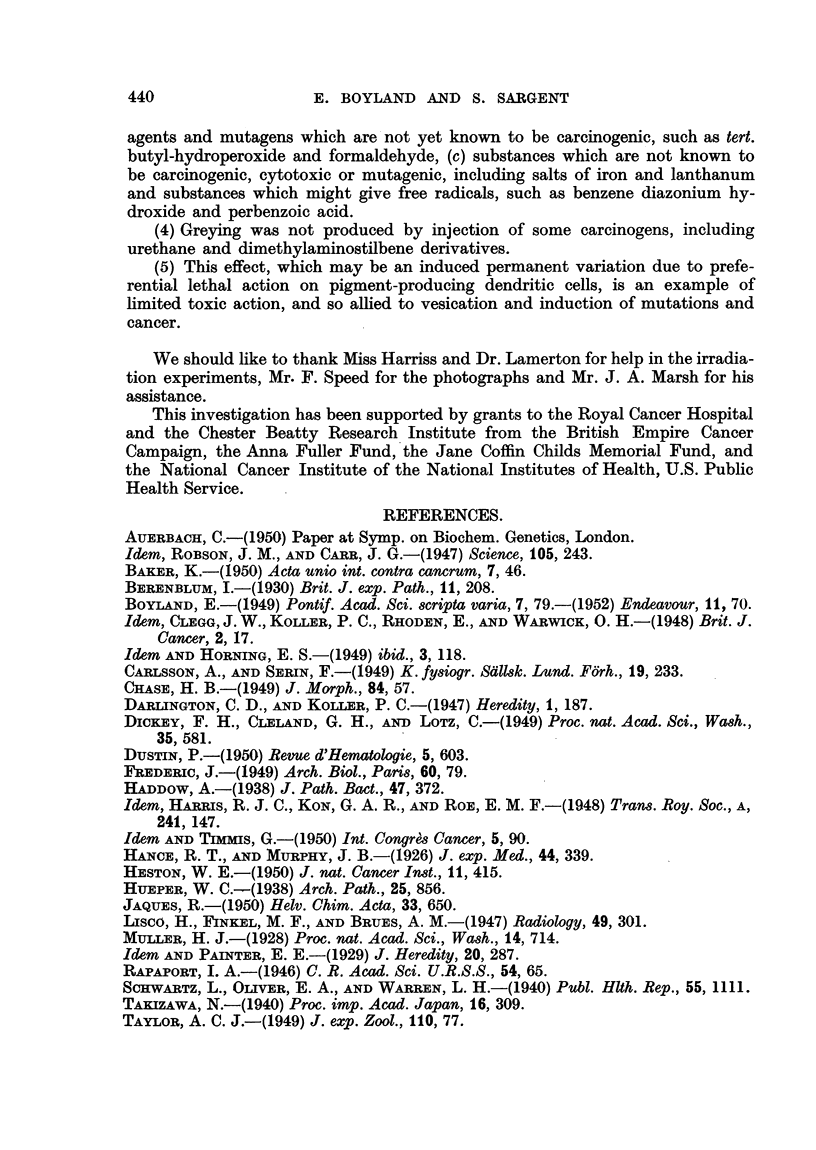


## References

[OCR_01143] Auerbach C., Robson J. M., Carr J. G. (1947). The Chemical Production of Mutations.. Science.

[OCR_01161] DICKEY F. H., CLELAND G. H., LOTZ C. (1949). The role of organic peroxides in the induction of mutations.. Proc Natl Acad Sci U S A.

[OCR_01163] DUSTIN P. (1950). Lésions cellulaires provoquées par les acides 4-aminoptéroylglutamiques chez la souris.. Rev Hematol.

[OCR_01174] HESTON W. E. (1950). Carcinogenic action of the mustards.. J Natl Cancer Inst.

[OCR_01180] Muller H. J. (1928). The Production of Mutations by X-Rays.. Proc Natl Acad Sci U S A.

